# Twin fetus in fetu in a child: a case report and review of the literature

**DOI:** 10.1186/1752-1947-4-96

**Published:** 2010-03-25

**Authors:** Ajay N Gangopadhyay, Arvind Srivastava, Punit Srivastava, Dinesh K Gupta, Shiv P Sharma, Vijayendra Kumar

**Affiliations:** 1 Department of Pediatric Surgery, Institute of Medical Sciences, Banaras Hindu University, Varanasi, India; 2 Department of Radiodiagnosis, Institute of Medical Sciences, Banaras Hindu University, Varanasi, India

## Abstract

**Introduction:**

Fetus in fetu is an extremely rare condition wherein a malformed fetus is found in the abdomen of its twin. This entity is differentiated from teratoma by its embryological origin, its unusual location in the retroperitoneal space, and the presence of vertebral organization with limb buds and well-developed organ systems. The literature cites less than 100 cases worldwide of twin fetus in fetu.

**Case presentation:**

A two-and-a-half-month-old Asian Indian baby boy had two malformed fetuses in his abdomen. The pre-operative diagnosis was made by performing an ultrasound and a 64-slice computer tomography scan of the baby's abdomen. Two fetoid-like masses were successfully excised from the retroperitoneal area of his abdomen. A macroscopic examination, an X-ray of the specimen after operation, and the histological features observed were suggestive of twin fetus in fetu.

**Conclusion:**

Fetus in fetu is an extremely rare condition. Before any operation is carried out on a patient, imaging studies should first be conducted to differentiate this condition from teratoma. Surgical excision is a curative procedure, and a macroscopic examination of the sac should be done after twin or multiple fetus in fetu are excised.

## Introduction

Fetus in fetu (FIF) is a rare condition associated with abnormal embryogenesis in a diamniotic, monochorionic pregnancy, wherein a vertebrate fetus is enclosed within the body of another normally developing fetus [1]. The FIF complex is characteristically composed of a fibrous membrane (equivalent to the chorioamniotic complex) that contains some fluids (equivalent to the amniotic fluid) and a fetus suspended by a cord or pedicle. In the uterus, the growth of an FIF initially parallels that of its twin, but stops abruptly because of either the vascular dominance of the host twin or an inherent defect in the parasitic twin [2]. FIF is mostly anencephalic, but in almost all cases its vertebral column and limbs are present (91% and 82.5%, respectively). At the same time its lower limbs are more developed than the upper limbs.

An FIF is rarely found in the central nervous system, gastrointestinal tract, retroperitoneum, vessels or genitourinary tract of its host twin. It is found even more rarely in the lungs, adrenal glands, pancreas, spleen or lymph nodes [3]. Even without performing an operation to remove the parasitic twin, the existence of the condition can be diagnosed through ultrasonography, plain X-ray and a computed tomography (CT) scan of the host's abdomen. The surgical removal of the twin fetus is the treatment of choice.

In most cases of FIF, only one fetus exists inside the baby. Only in extremely rare cases are multiple fetuses found.

## Case presentation

**Figure 1:**
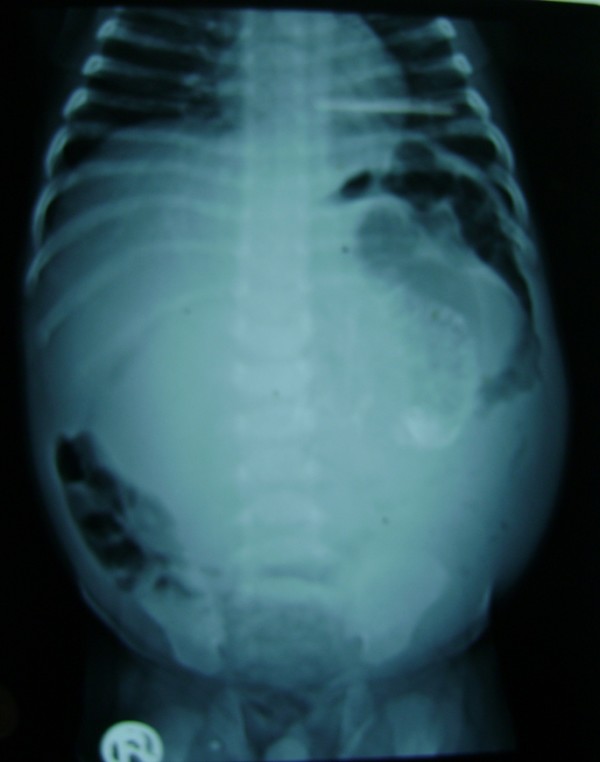
**Plain X-ray of the vertical calcification on the left side of the abdomen**.

**Figure 2:**
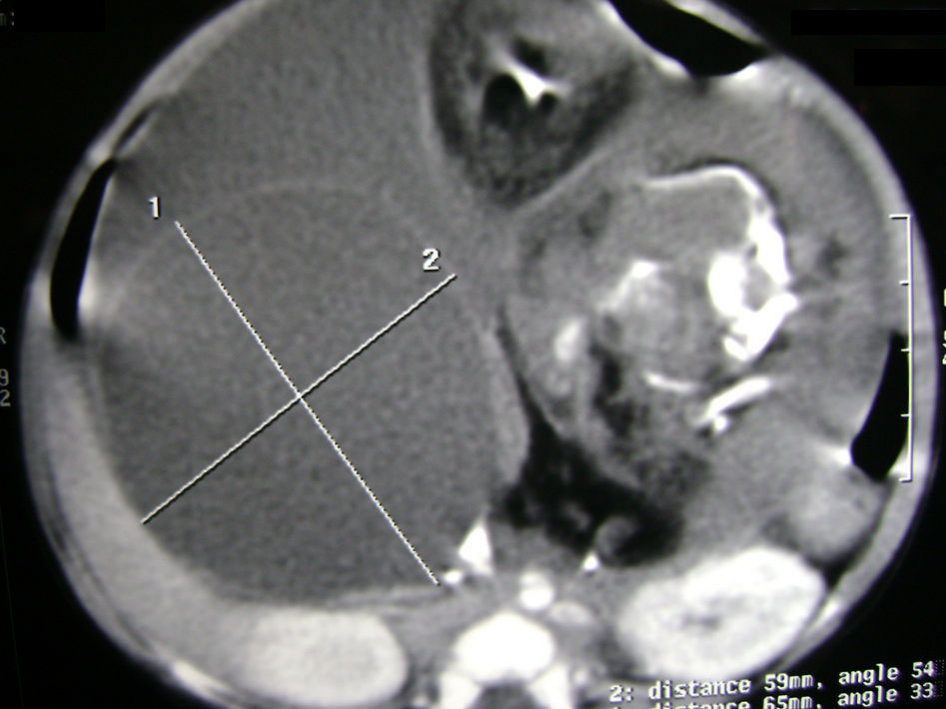
**Abdominal computed tomography of the fetus with a large encapsulated peritoneal cavity mass and mature vertebral skeleton**.

**Figure 3:**
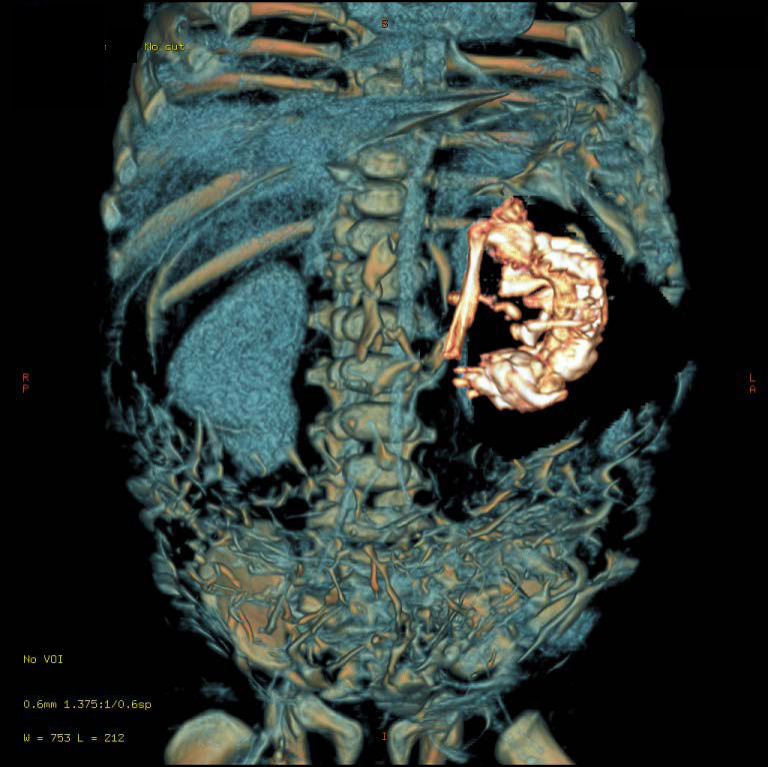
**A 64-slice computed tomography scan of the bony outline of the fetus in fetu**.

A two-and-a-half-month-old, first-born, Asian Indian baby boy was admitted to the department of Pediatric surgery, S.S hospital, BHU, due to recurrent episodes of vomiting and abdominal distension since he was one month old. Upon examination of the baby's abdomen we discovered that a smooth, firm and non-tender mass was present in the left half of his abdomen. Conventional X-ray of the abdomen showed a soft tissue mass with a vertebrae-like column (Figure 1). An ultrasound of the baby's abdomen showed a large, encysted, hyperechoic and calcified heterogenous complex mass. A 64-slice CT scan of his abdomen revealed a soft tissue mass that had a bony outline resembling a fetus (Figures 2 and 3). Interestingly, we found nothing significant in the baby's family history.

**Figure 4:**
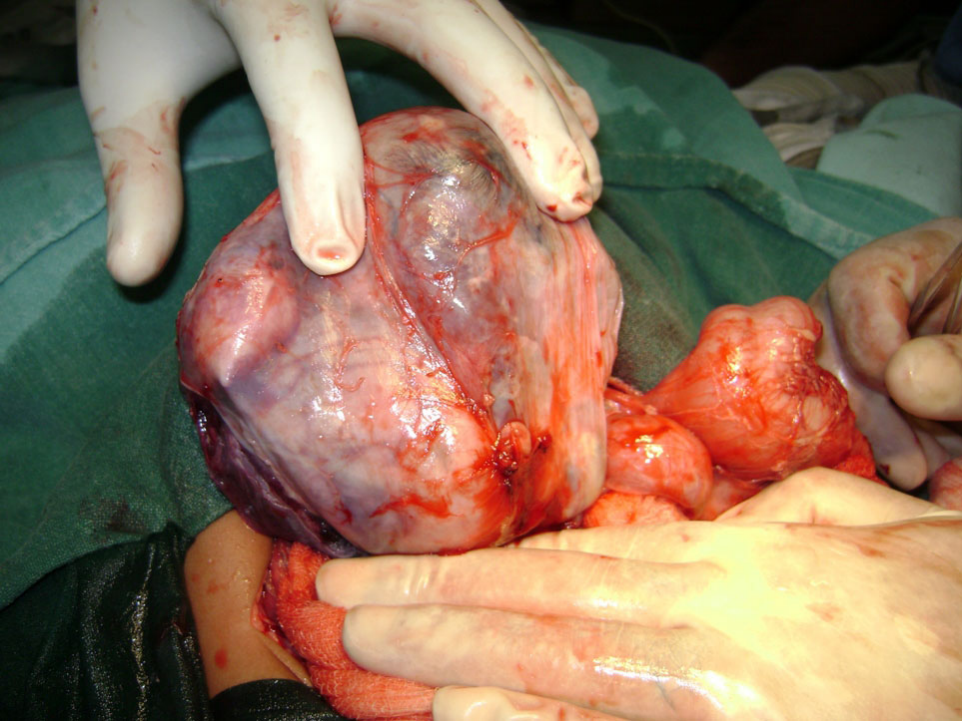
**Intra-operative picture of the fetus in fetu enveloped by a sac**.

We performed an elective laparotomy after correcting the baby's fluid and electrolyte levels. We then found a well-encapsulated cystic retroperitoneal mass that was displacing his spleen, transverse colon and pancreas. This displacement presented laterally and caudally toward his cephaloid and left kidney (Figure 4). The mass had a separate blood supply connected to the baby's abdominal aorta just below his left renal artery. We mobilized, without complication, his left colon, pancreas, duodenum and small bowel, after which we were able to excise the mass completely.

**Figure 5:**
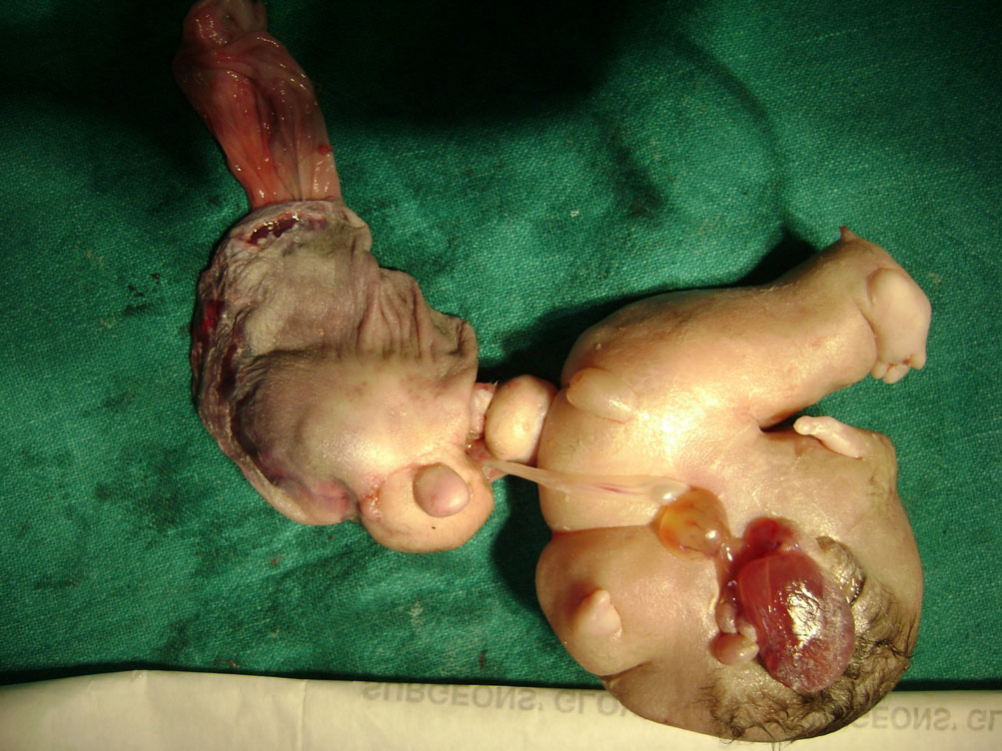
**Twin fetus in fetu connected by a cord-like structure**.

**Figure 6:**
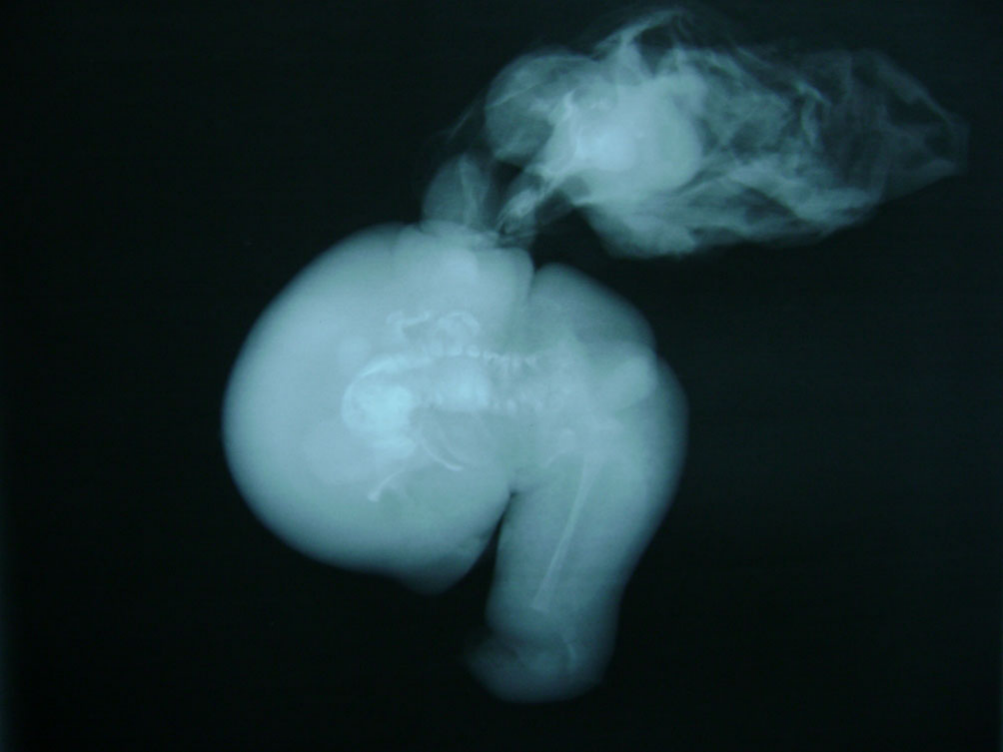
**Plain X-ray of the fetal specimen with a vertebral column**.

The sac contained two miniature fetuses connected to each other by a cord-like structure at the umbilicus. The miniature fetuses had a well-defined foot, skin with hairs, a convex and pliable skull bone, and other undifferentiated tissues (Figure 5). A radiograph of the specimen showed cranial bones and long bones with vertebral columns (Figure 6). We then performed a macroscopic pathological examination, from which we were able to note that the mass measured 20 × 8 × 5 cm. It was also composed of a head with hair, a trunk, and rudimentary limbs connected by cord-like structures. The mass corresponded to an incompletely developed twin fetus.

A microscopic examination showed that the underdeveloped twin had mature embryonic tissues containing elements of the three germinative layers. Skin, a vertebral column, germinative buds of limbs, central nervous tissue (encephalus and coroidal plexus), a stomach, small and large bowels, pancreas, adrenal glands, kidneys, upper and lower airways, cardiac striated muscles, and lymphoid tissue-like spleen were found. The histopathological study of the specimen supported the conclusion that the previously imaged calcifications could be assumed to be the skull and bony constituents of the vertebral axis, some parts of the skull, and bony constituents of the rudimentary limbs.

Our patient recovered well after the surgery and was discharged. To rule out any recurrence he was followed up through clinical examination, plain abdominal X-ray examination, abdominal ultrasound, and serum alpha-fetoprotein (AFP). We were unable to detect any recurrence of his previous symptoms one year after the operation.

## Discussion

The term "fetus in fetu" was first used by Johann Friedrich Meckel during the late 18th century [4]. Subsequently, Willis described it as a rare condition where a malformed parasitic twin resides in the body of its host, usually in the host's abdominal cavity [5]. The condition represents an aberration of monozygotic diamniotic twinning where the unequal division of the totipotent inner cell mass of the developing blastocyst leads to the inclusion of a smaller cell mass within a maturing sibling embryo.

This rare pathology occurs only once every 500,000 births [6]. Fewer than 100 cases worldwide have been reported [7]. The literature rarely describes multiple or twin FIF. The majority of cases of FIF occur during infancy, with the oldest reported case being that of a 47-year-old man [1]. Thakral *et al. *reported that FIF occurs equally among the male and female populations [8]. In 70% of reported cases, the chief presenting complaint is an abdominal mass [9]. The mass is predominantly retroperitoneal in 80% of cases [5], while reported uncommon sites are the oral cavity [4], the sacrococcygeal region [10] and the scrotum [7].

The presence of a vertebral column in the FIF is an important feature that distinguishes it from a teratoma. The clear identification of a verterbal column shows that fetal development of the included twin had advanced at least beyond the primitive streak stage (12 to 15 days of gestation) to a notochord, which is the precursor of the vertebral column [1-3,8]. FIF generally occurs singly. Multiple masses have been found in only a few instances. Our patient exemplifies the occurrence of FIF as a partially developed twin fetus [11]. The mass we found in our patient was enveloped by a sac that contained a second mass, which was suggestive of a twin FIF. There are instances where no symptoms at all occur. In some cases, however, symptoms present as an effect of the mass, such as abdominal distension, feeding difficulties, emesis, jaundice and dyspnea [2,11]. In our case, our patient presented with distension of the abdomen and recurrent vomiting.

The pre-operative diagnosis of FIF depends on its related radiological findings. Plain abdominal X-ray examination may prove helpful, as up to half of reported cases show the presence of a vertebral column and axial skeleton [1], which was also the case for our patient. Meanwhile, Hoeffel *et al. *[1] discussed the inability of radiographic examination to visualize the vertebral axis of the FIF. This inability to visualize the vertebral axis when a patient is examined through a CT scan, however, should not lead to diagnostic exclusion because an under-developed and markedly dysplastic spinal column may have prevented identification of the pathology at imaging.

Sonographic findings are usually those of a complex cystic mass with ill-defined solid internal components. Imaging continues to play an important role in diagnosing FIF. CT and MRI have been proven to be very helpful in suggesting a pre-operative diagnosis [11]. In our case, diagnosis was made pre-operatively through a CT scan; nevertheless abdominal ultrasound cannot be ignored in the initial evaluation of the anatomy of FIF. The twin fetus is typically suspended by a pedicle within a complete sac that contains fluid or sebaceous material. There is no placenta or chorionic villi at the point of attachment to the host [12]. In our case, our patient's twin was present within the sac in his left retroperitoneum. The twin was also found suspended by a vascular pedicle to its host's abdominal aorta, and multiple vascular attachments to the surrounding bowel were noted. It is important to note that the presence of a twin fetus in the host's abdomen is extremely rare.

Although the prognosis for FIF is more favorable than for cystic teratoma, the presence of immature elements nevertheless indicates the need for close clinical, radiological and serological (AFP) follow-up [6]. Despite the AFP levels before and after surgery remaining at normal values, a possible recurrence of a malignant teratoma after FIF resection must best be kept in mind. This was the reason why we continued to monitor the serial tumor marker levels of our patient, while also conducting cross-sectional imaging follow-up examinations [1].

## Conclusion

Alhtough it is rarely the conclusive diagnosis, FIF should still be considered in a child presenting with progressively increasing abdominal swelling and vomiting. Although definitive diagnosis is best made using CT and MRI techniques, plain X-rays and ultrasonography can still be useful in the initial work-up prior to surgery. Post-operative X-ray examination of a specimen from the mass can ultimately confirm the diagnosis of FIF. The mass, however, should still be examined for the occurrence of multiple fetus even after it has already been excised.

## Consent

Written informed consent was obtained from the parents of our patient for publication of this case report and any accompanying images. A copy of the written consent is available for review by the Editor-in-Chief of this journal.

## Competing interests

The authors declare that they have no competing interests.

## Authors' contributions

ANG, SPS and PS operated on our patient and reviewed the literature. DKG and VK were the main moderators of the manuscript. All authors read and approved the final manuscript.
